# Promising Antioxidant and Anticorrosion Activities of Mild Steel in 1.0 M Hydrochloric Acid Solution by *Withania frutescens* L. Essential Oil

**DOI:** 10.3389/fchem.2021.739273

**Published:** 2021-10-11

**Authors:** Abdelfattah El moussaoui, Mariya Kadiri, Mohammed Bourhia, Abdelkrim Agour, Ahmad Mohammad Salamatullah, Abdulhakeem Alzahrani, Heba Khalil Alyahya, Nawal A. Albadr, Mohamed Chedadi, Mouhcine Sfaira, Amina Bari

**Affiliations:** ^1^ Laboratory of Biotechnology, Environment, Agrifood, and Health, Faculty of Sciences, Sidi Mohamed Ben Abdellah University (USMBA), Fez, Morocco; ^2^ Laboratory of Engineering, Modeling and Systems Analysis (LIMAS), Faculty of Sciences, Sidi Mohamed Ben Abdellah University (USMBA), Fez, Morocco; ^3^ Laboratory of Chemistry-Biochemistry, Environment, Nutrition, and Health, Faculty of Medicine and Pharmacy, Hassan II University, Casablanca, Morocco; ^4^ Laboratory of Natural Substances, Pharmacology, Environment, Modeling, Health and Quality of Life, Faculty of Sciences, Sidi Mohamed Ben Abdellah University (USMBA), Fez, Morocco; ^5^ Department of Food Science and Nutrition, College of Food and Agricultural Sciences, King Saud University, Riyadh, Saudi Arabia

**Keywords:** *Withania frutescens L*, essential oil, antioxidant, anti-corrosion, chemical composition

## Abstract

The present study was conducted to evaluate the anticorrosive and antioxidant activities of essential oil from *Withania frutescens* L. In the present study, the extraction of *Withania frutescens* L. essential oil (Wf-EO) was conducted using hydrodistillation before being characterized by gas chromatographic analysis (GC/MS) and flame ionization detector (GC/FID). Four bioassays were used for antioxidant testing including 2,2-diphenyl-1-picrylhydrazyl (DPPH), total antioxidant capacity (TAC), ferric reducing antioxidant power (FRAP), and β-carotene bleaching. The inhibiting effect of Wf-EO on the corrosion behavior of mild steel in 1.0 M HCl was conducted by using polarization curves and electrochemical impedance spectroscopy techniques. The yield of Wf-EO was 0.46% including 175 compounds identified by GC-MS. The oil was mostly constituted of camphor (37.86%), followed by thujone (26.47%), carvacrol (6.84%), eucalyptol (3.18%), and linalool (2.20%). The anti–free radical activity of Wf-EO was 34.41 ± 0.91 μg/ml (DPPH), 9.67 ± 0.15 mg/ml (FRAP), 3.78 ± 0.41 mg AAE/g (TAC), and 89.94 ± 1.44% (β-carotene). The Wf-EO showed potent antioxidant activity in all bioassays used for testing. The anticorrosion activity, polarization curves as well as EIS diagrams indicated that the Wf-EO exhibited anticorrosive properties and reacted as a suitable corrosion inhibitor in an acidic medium.

## Introduction

Corrosion is a natural process that involves interactions between a material and its environment leading to changes in the properties and characteristics of the metal resulting in alteration of the material (DIN EN ISO 8044, 2020). Corrosion has been considered among the problems of most industrial sectors, and it can cost many billions of dollars every year due to deterioration of materials (Hussin and Kassim, 2011). Exposed metals to certain acids like HCl can accelerate the corrosion process since such acids act as corrosive agents. The corrosive agents have an important role in petrochemical processes, industry, refining of crude oil, industrial cleaning, and acid descaling (Abiola and James, 2010).

Hydrochloric acid (HCl) is among the most widely used corrosive agents in the industrial field. HCl causes the degradation of metals through electrochemical and/or chemical reactions. To solve such corrosion problems, there are some methods to protect metals from corrosion from acidic environments including cathodic protection, use of protective barriers, use of anti-rust solutions or corrosion inhibitors, and galvanization. Substances, reacting as corrosion inhibitors, can reduce or prevent the reaction of the metal in the acidic environment (Abdel-Gaber et al., 2006; Njong et al., 2018).

Most of the synthetic or semi-synthetic compounds possess good anticorrosive activity, but some chemicals that are applied for corrosion inhibition may be harmful to humans (Aourabi et al., 2020). It is thus fitting that the interest in natural anticorrosive agents has grown worldwide. In this sense, Wf-EO can be one of the preferable agents that find application in the industrial sector to fight against corrosion (Lahhit et al., 2011; Gualdrón et al., 2013).

In the framework of seeking potential natural products for use against corrosion, this work was conducted to test Wf-EO, which is known by its therapeutic applications particularly antimicrobial, anti-diabetic, anti-inflammatory, and healing activities (EL Moussaoui et al., 2019a; EL Moussaoui et al., 2019b; El Moussaoui et al., 2020a, Moussaoui et al., 2021 A.; EL Moussaoui A. E. et al., 2021). Moreover, no *in vivo* toxicities have been reported for Wf-EO at doses up to 2000 mg/kg/weight (EL Moussaoui et al., 2020b; Moussaoui et al., 2020).

To the best of our knowledge, no works have reported the anticorrosive activity of *Withania frutescens* L. essential oil (Wf-EO) up to date. Therefore, the present work aimed at the chemical composition study, antioxidant, and anticorrosive activities of Wf-EO leaves. The anticorrosion activity was undertaken using potentiodynamic polarization (PP) and electrochemical impedance spectroscopy (EIS) methods.

## Material and Method

### Extraction of *Withania frutescens* L. Essential Oil

*Withania frutescens* L. was collected in March when the vegetation and flowering was at its peak. Next, the plant was identified by a botanist and given a number BPRN-69 before being deposited at the herbarium. Afterward, the leaves of the studied plant were dried at 35°C using an oven. Later, the aerial parts of *Withania frutescens* L. were ground into a fine powder using an electric mill. The essential oil of the obtained powder was extracted by hydro-distillation. Thereafter, the Wf-EO was recovered and kept at 4°C until further use.

### Gas Chromatography Analysis of *Withania frutescens* L. Essential Oil

GC-FID (Shimadzu, Kyoto, Japan) was used to perform the analysis. GC2010 system equipped with an SLB-5 ms fused silica capillary column with 30 m × 0.25 mm i.d. × 0.25 μm df. In this analysis, helium was used as a carrier gas (flow rate = 30.0 cm/s). The sample was meticulously dissolved in ethyl acetate solvent (10% w/w). Next, 0.5 μl of the studied sample was injected for analysis (the split ratio was 1:20). The oven temperature was standardized at 50°C/3 min and then 350°C/5 min. The injection temperature was adjusted at 280°C (the rate was of 200 ms). The hydrogen and airflow rates were 40 ml/min and 400 ml/min, respectively. Data processing was performed by LabSolution software ver. 5.92. The identification of the constituents was conducted by using two filters, namely linear retention index (LRI) and spectral similarity match over 85%. Kovats index was calculated using series of alkanes, from C7 to C40 1,000 g/ml, 49452-U from Merck KGaA (Darmstadt, Germany) with a filter design ±10 LRI units. The obtained mass spectra were compared with those reported in the literature including W11N17 (DB1) (Mass Finder 3 (DB2) (D.H. Hochmuth, www.massfinder.com; Wiley11-Nist17, Wiley, and FFNSC 3.0 (DB3); Hoboken, United States) (Schnitzler et al., 1998; Kelsey, 1999; Adams, 2007; Hattab et al., 2007; Dimou et al., 2016).

### *In vitro* Antioxidant Activity of *Withania frutescens* L. Essential Oil

The investigation of the antioxidant property of Wf-EO was assessed *in vitro* using different assays including 2,2-diphenyl-1-picrylhydrazyl assay (DPPH), reducing power (FRAP), β-carotene bleaching, and total antioxidant capacity (TAC).

#### 2,2-Diphenyl-1-picrylhydrazyl Assay

The antioxidant activity by the DPPH test was performed following the method described by BEKTAS with a few modifications (Moussaoui et al., 2021). Briefly, different concertinos of Wf-EO were mixed with 750 µl of DPPH (4.10^−2^ mg/ml). After incubation at ambient temperature for 30 min, the reading of absorbance was conducted at 517 nm. The results were expressed as percentage inhibition according to the following formula:

PI(%)=(A0−A/A0)∗100, (1)

where PI is percentage of inhibition; A_0_ is absorbance of the DPPH solution (negative control); and A is absorbance of the DPPH solution mixed with sample.

#### Ferric Reducing Antioxidant Power Test

The FRAP test was performed according to Moattar’s method. In summary, 500 µl of a buffer phosphate solution with 0.2 M, pH = 6.6, and 500 µl of potassium ferricyanide [K_3_Fe(CN)_6_] (10 mg/ml) were mixed with 100 µl of different concentrations of Wf-EO. After incubation in a water bath (50°C) for 20 min, 500 µl of both aqueous TCA solution (10%), and distilled water, and 100 µl FeCl_3_ (0.1%) were supplemented to the reaction medium. Next, the absorbance of the sample (Wf-EO) was measured at 700 nm, and the results were expressed as half-maximal effective concentration (EC-50) (EL Moussaoui et al., 2019c).

#### Total Antioxidant Capacity Test

Twenty-five microliters of the Wf-EO were mixed with reagent solution including sulfuric acid (0.6 M), sodium phosphate (28 mM), and ammonium molybdate (4 mM). Afterward, the reaction medium was incubated at 95°C for 90 min. The absorbance of the solution was measured at 695 nm using a spectrophotometer). The results were expressed in mg EAA/g Wf-EO (EL Moussaoui et al., 2019c).

#### Beta-Carotene Discoloration Test

β-carotene (1 ml) was mixed with chloroform (0.2 mg/ml), and then the whole was then mixed with 10 µl linoleic acid and Tween 80 (100 mg). Next, the chloroform solvent was evaporated under reduced pressure at 45°C and 25 ml of hydrogen peroxide (H_2_O_2_) was added to the reaction medium. The resulting mixture (2.5 ml) was mixed with 0.1 ml of the diluted Wf-EO and then incubated at 50°C for 120 min with BHT reagent. The antioxidant percentage was calculated by the following formula:

AA%=(AE/ABHT)×100, (2)

where AA% is the antioxidant activity percentage, AE is the absorbance of the sample, and ABHT is the absorbance of the sample without BHT.

### The Anticorrosion Activity of *Withania frutescens* L. Essential Oil

#### Material

Mild steel (MS) with following chemical composition: 0.3700% C, 0.1600% Cu, 0.2300% Si, 0.6800% Mn, 0.0770% Cr, 0.0160% S, 0.0090% Co, 0.0110% Ti, 0.0590% Ni, and the remainder iron (Fe) was used as a substrate for anticorrosion activity testing. Before all measurements, the sample was abraded using emery papers, rinsed with water before being degreased in acetone, and finally washed again using distilled water.

#### Corrosive Medium

The electrolyte was obtained through dilution of hydrochloric acid (37%) (*d* = 1.18). In this sense, Wf-EO was previously dissolved in 10 ml of ethanol bi-distilled before being added to 90 ml of 1.0 M HCl. The concentrations used in this work ranged from 1 g L^−1^ to 0.1 g L^−1^.

#### Electrochemical Measurements

Electrochemical measurements were performed using the BioLogic SP-150 device controlled with software (Ec-Lab® V11.200). All experiments were conducted in the conventional three-electrode cell with MS described earlier as a serving electrode, platinum and Ag/AgCl 3M KCl were used as reference and counter electrodes, respectively. For the potentiodynamic polarization (PP) measurements, the potential was swept from −250 to **+**250 *mV* at a scan rate of 1 *mV s* ^*−1*^ relative to the potential were swept. Electrochemical impedance spectroscopy (EIS) measurements were carried out after a steady-state was achieved in a frequency ranging from 100.00 *kHz* to 100.00 *mHz* with ten points per decade and AC voltage amplitude of 10 *mV* peak to peak. Before each measurement, the electrode was kept at *E*_*corr*_ for 30 min at a temperature of 298 K and the test was repeated twice*.*

### Statistical Analysis

Mean values of results alongside standard deviations were calculated by GraphPad Prism software (Microsoft Soft-ware). For comparison purposes, one-way ANOVA and Tukey’s test were used to achieve this goal. Differences at *p* < 0.05 were considered significant.

## Results and Discussion

### Phytochemical Identification of *Withania frutescens* L. Essential Oil

**FIGURE 1:**
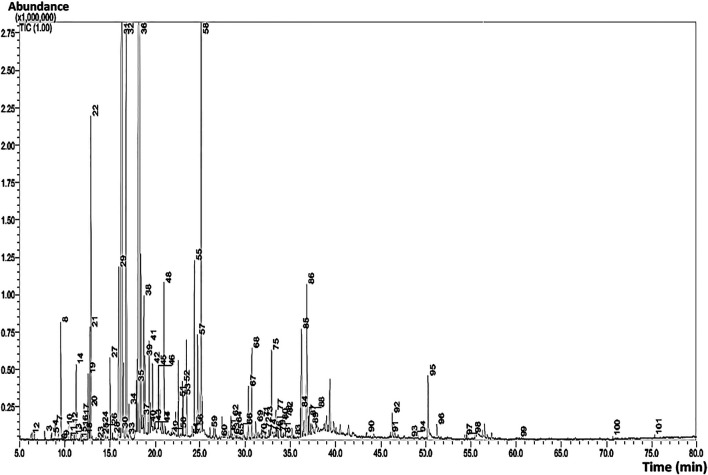
GC/MS chromatogram of the characterized Wf-EO.

**FIGURE 2:**
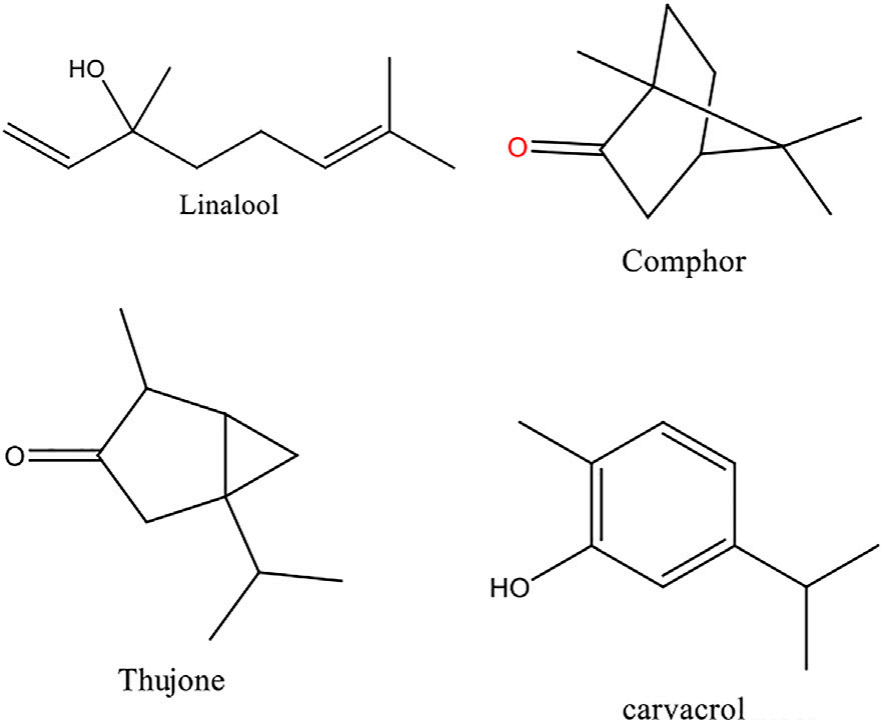
Molecular structure of the main compounds identified in the Wf-EO.

The results of GC-MS analyses of Wf-EO are presented in Figure 1 and Table 1. The obtained results showed that the extraction yield of essential oil recovered from *Withania frutescens* L was 0.46% with 175 compounds. The significant peaks displayed on the GC-MS chromatogram corresponded to major compounds in the oil, such as camphor (55), β-thujone (46), carvacrol (94), β-thujone (48), eucalyptol (32), and linalool (44) (Figure 2). Carvacrol (5-isopropyl-2-methylphenol) that previously reported in the family Lamiaceae including genera *Origanum* and *Thymus* possessed anti-inflammatory and antimicrobial properties (Lima et al., 2013). Eucalyptol identified in genus *Eucalyptus* showed antioxidant, antimicrobial, antiseptic, anti-inflammatory, and anti-parasitic activities (Salhi, 2014; Aleksic Sabo and Knezevic, 2019). Camphor found in *Lavandula officinalis* and *Cinnamomum camphora* had antioxidant and anti-inflammatory activities (Lee et al., 2006; Barkat and Imène, 2012). Camphor is also found in other plants including *Rosemary* essential oil, which has been used in pharmaceutical and cosmetic industries (Gonçalves et al., 2020). Alpha and beta thujone are detected in the oil of several plants including *Salvia officinalis* and *Artemisia herba alba*, which have been used in perfume, pharmaceutical, cosmetics, and food industries. Linalool identified in *Lavandula officinalis* and *Juniperus phoenicea,* exhibited antioxidant, antimicrobial, and insecticidal activities (Bouzouita et al., 2008; Barkat and Imène, 2012).

**TABLE 1:** Constituents identified in the Wf-EO by GC/MS analysis.

N°	Compound	**% MS Sim**	LRI (Ref)	LRI (Exp)	Area%
**1**	(Z)-Salvene	95	846	846	0,02
**2**	Hex-(3Z)-enol	94	853	851	0,01
**4**	Santolinatriene	95	902	902	0,04
**6**	Artemisia triene	87	922	921	0,04
**7**	Tricyclene	94	923	923	0,01
**8**	α-Thujene	93	927	927	0,09
**9**	α-Pinene	97	933	933	0,11
**11**	Camphene	96	953	949	0,80
**13**	Thuja-2,4(10)-diene	90	953	953	0,08
**14**	Benzaldehyde	93	960	963	0,07
**15**	Sabinene	93	972	972	0,02
**16**	β-Pinene	91	978	978	0,02
**19**	Myrcene	94	991	988	0,02
**21**	Yomogi alcohol	97	996	995	0,47
**22**	*n-*Octanal	90	1,006	1,004	0,18
**23**	α-Phellandrene	97	1,007	1,007	0,04
**24**	δ-3-Carene	96	1,009	1,010	0,04
**25**	α-Terpinene	95	1,018	1,017	0,11
**27**	*p-*Cymene	96	1,025	1,025	0,29
**30**	Limonene	90	1,030	1,029	0,06
**31**	Santolina alcohol	93	1,033	1,031	0,46
**32**	Eucalyptol	91	1,032	1,032	3,18
**33**	(E)-, β-Ocimene	87	1,046	1,046	0,30
**35**	Artemisia ketone	93	1,056	1,057	0,02
**36**	γ-Terpinene	92	1,058	1,058	0,03
**38**	*n-*Octanol	90	1,076	1,071	0,04
**40**	Artemisia alcohol	96	1,079	1,080	0,52
**42**	Camphenilone	90	1,084	1,085	0,05
**44**	Linalool	98	1,101	1,101	2,20
**45**	*n-*Nonanal	93	1,107	1,106	0,09
**46**	β-Thujone	96	1,118	1,109	26,47
**48**	α-Thujone	98	1,118	1,120	6,36
**49**	Dehydro-Sabina ketone	91	1,122	1,122	0,03
**50**	(Z), *p-*Menth-2-en-1-ol	98	1,124	1,126	0,22
**53**	(E)-Pinocarveol	97	1,141	1,144	0,25
**55**	Camphor	97	1,149	1,151	37,86
**57**	Camphene hydrate	93	1,156	1,157	0,36
**59**	Santolinyl acetate	94	1,175	1,161	0,79
**60**	Pinocarvone	97	1,164	1,164	0,75
**61**	δ-Terpineol	90	1,170	1,172	0,19
**62**	Borneol	95	1,173	1,174	0,59
**65**	Terpinen-4-ol	92	1,184	1,182	0,48
**68**	*p-*Cymen-8-ol	90	1,189	1,189	0,08
**69**	α-Terpineol	89	1,195	1,197	0,23
**70**	Myrtenal	91	1,197	1,198	0,46
**72**	*n-*Decanal	90	1,208	1,206	0,03
**73**	Octyl-acetate	95	1,214	1,210	0,66
**76**	3-Isopropyl-phenol	90	1,228	1,227	0,01
**77**	Bornyl formate	96	1,230	1,230	0,03
**79**	Cuminaldehyde	95	1,243	1,245	0,02
**83**	Piperitone	93	1,267	1,256	0,25
**84**	(Z)-Chrysanthenyl acetate	89	1,257	1,258	0,27
**86**	Perillaldehyde	87	1,278	1,278	0,00
**88**	Bornyl acetate	98	1,285	1,285	0,94
**90**	(E)-Sabinyl acetate	90	1,291	1,289	0,06
**92**	Thymol	97	1,293	1,293	0,56
**94**	Carvacrol	95	1,300	1,302	6,84
**96**	Myrtenyl acetate	88	1,324	1,324	0,06
**99**	α-Cubebene	87	1,347	1,348	0,06
**102**	Cyclosativene	94	1,367	1,371	0,05
**105**	α-Copaene	93	1,375	1,377	0,11
**106**	(E)-, β-Damascenone	94	1,379	1,380	0,04
**107**	β-Bourbonene	95	1,382	1,386	0,05
**109**	β-Elemene	92	1,390	1,391	0,02
**111**	decyl-Acetate	93	1,412	1,408	0,05
**112**	(E)-Caryophyllene	97	1,424	1,422	0,39
**113**	γ-Elemene	94	1,432	1,431	0,60
**114**	Aromadendrene	96	1,438	1,441	0,10
**116**	Isogermacrene D	90	1,447	1,451	0,03
**117**	α-Humulene	96	1,454	1,458	0,04
**118**	9-*epi-*(E)-Caryophyllene	90	1,464	1,463	0,05
**119**	γ-Decalactone	95	1,469	1,467	0,02
**120**	Selina-4,11-diene	91	1,476	1,475	0,05
**123**	Germacrene D	95	1,480	1,483	0,58
**125**	β-Selinene	89	1,492	1,491	0,03
**126**	Valencene	92	1,492	1,495	0,13
**128**	Bicyclogermacrene	92	1,497	1,498	0,13
**129**	ε-Amorphene	89	1,502	1,501	0,04
**132**	β-Bisabolene	89	1,508	1,508	0,03
**133**	γ-Cadinene	89	1,512	1,516	0,03
**134**	δ-Cadinene	93	1,518	1,521	0,16
**138**	α-Calacorene	89	1,544	1,544	0,01
**139**	Germacrene B	90	1,557	1,562	0,34
**140**	*n-*Dodecanoic acid	96	1,581	1,566	0,42
**141**	Spathulenol	94	1,576	1,581	0,31
**142**	Caryophyllene oxide	90	1,587	1,586	0,15
**143**	Thujopsan-2-p-ol	95	1,589	1,588	0,20
**144**	ethyl-Dodecanoate	90	1,598	1,593	0,07
**157**	*n-*Tetradecanoic acid	90	1773	1760	0,01
**158**	Neophytadiene	93	1836	1835	0,01
**159**	Phytone	95	1841	1841	0,13
**161**	*n-*Nonadecane	89	1900	1899	0,01
**162**	methyl-Hexadecanoate	93	1925	1925	0,04
**163**	*n-*Hexadecanoic acid	94	1977	1963	0,06
**164**	ethyl-Palmitate	93	1993	1992	0,01
**165**	methyl-Linoleate	90	2093	2092	0,03
**166**	methyl-Linolenate	89	2098	2098	0,04
**173**	*n-*Tricosane	92	2,300	2,299	0,01
**174**	*n-*Heptacosane	93	2,700	2,699	0,02
**175**	*n-*Nonacosane	90	2,900	2,898	0,02

### *In vitro* Antioxidant Activities of *Withania frutescens* L. Essential Oil

**FIGURE 3:**
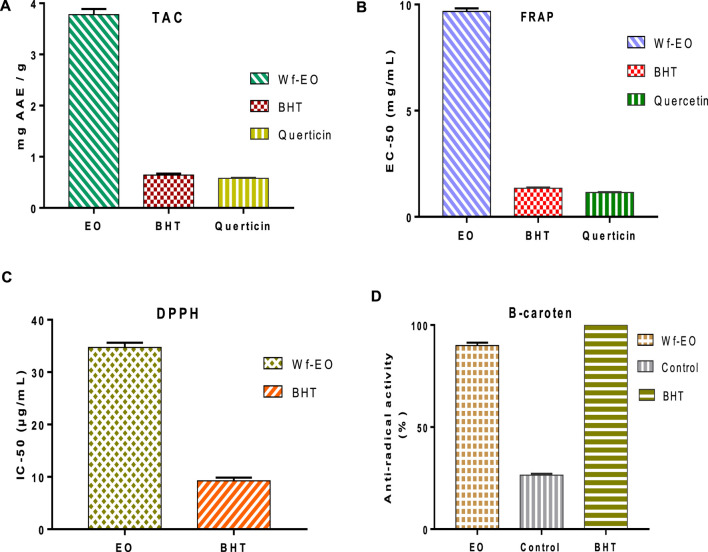
Antioxidant activity of Wf-EO essential oils by TAC **(A)**, FRAP **(B)**, DPPH **(C)**, and B-carotene **(D)**.

In the present research study, the antioxidant activity of *W. frutescens* oils was done by using four tests including DPPH, FRAP, total antioxidant activity, and β-carotene. The obtained results are summarized in Figure 3. It was reported that essential oil possessed several compounds with different chemical behavior, different chemical functional groups, and different polarities. Therefore, the evaluation of antioxidant potentiality needs multiple assays to validate the free radical scavenging capacity results. The total antioxidant capacity revealed that Wf-EO, Butylated hydroxytoluene (BHT), and Quercetin (Figure 3A) were of the order of 3.78 ± 0.41 mg AAE/g, 0.64 ± 0.030 mg AAE/g, and 0.58 ± 0.010 mg AAE/g, respectively. It is thus fitting that, the results of antioxidant activity by the second test (FRAP) showed that Wf-EO essential oils possessed Fe^3+^ reducing ability and their reducing power (FRAP) increased with increasing concentration (dose dependency), and the effective concentrations of 50% (EC-50) of Wf-EO, BHT, and Quercetin were in the range of 9.67 ± 0.15 mg/ml, 1.35 ± 0.03 mg/ml, and 1.14 ± 0.02 mg/ml (Figure 3B), respectively. Scientific studies have confirmed that the antioxidant activity by FRAP test is correlated to the presence of antioxidant compounds in essential oils along with antioxidant agents that exert an effect on free radical chains (Blažeković et al., 2010). The evaluation of the reducing power by DPPH test is evaluated by the comparison of IC-50 values. As shown in Figure 3C, the IC_50_ values of Wf-EO and BHT were 34.41 ± 0.91 μg/ml, and 9.24 ± 0.61 μg/ml, respectively. Therefore, these results are consistent with those investigated in the existing literature, which showed that closer genus possessed antioxidant power (Guruprasad et al., 2019).

The variation of β-carotene agent as a function of time (0, 25, 50, 75, 100, and 120 min) indicated that Wf-EO reacts toward the oxidation of linoleic acid (Figure 3D). In this sense, the obtained results indicated that the percentage of β-carotene bleaching inhibition in the presence of Wf-EO, negative control, and positive control (BHT) was in the range of 89.94 ± 1.44%, 26.43 ± 0.73, and 100%, respectively. These results were considered important and confirmed by recent researches on the phytochemical compounds of *Withania frutescens* (Soltaninejad and Shahidi, 2018; EL Moussaoui et al., 2019a). Many studies have established an important relationship between the chemical composition of the essential oil and its antioxidant activity, and it has been reported that the antioxidant activity of essential oils is related to their chemical compositions, particularly through the presence of compounds containing a hydroxyl function as well as terpene alcohols and phenolic compounds (Gulçin et al., 2004; Bouhdid et al., 2008; Fayed 2009; Zhuang et al., 2009). Moreover, according to the obtained results in the present work, the essential oils of *Withania frutescens* species exhibited an important antioxidant efficacy even at the lowest concentration tested, which was in accordance with previous works (EL Moussaoui et al., 2020a).

### The Anticorrosion Activity of *Withania frutescens* L. Essential Oil

#### Potentiodynamique Polarization Measurements

**FIGURE 4:**
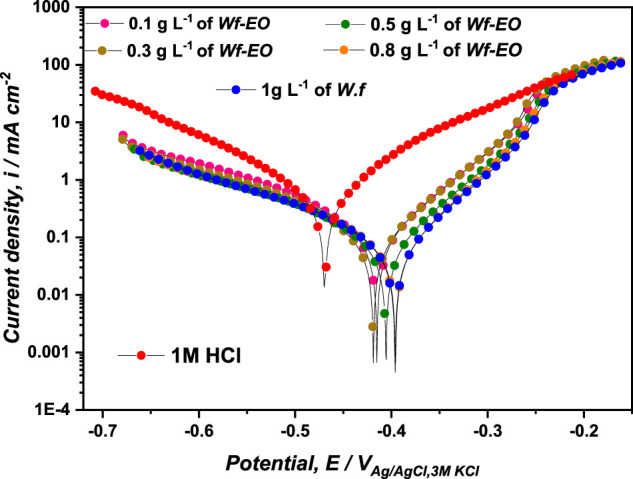
Polarization curves of MS in 1.0 M HCl at different concentrations of Wf-EO at 298 K.

PP measurements are a fundamental technique used to collect information related to an electrochemical system. The perturbations of MS in 1.0 M HCl medium, in the absence and presence of Wf-EO, were carried out after 30 min of stabilization at the corrosion-free potential called open circuit potential of the electrochemical system at 298 K. As reported in Figure 4. The electrochemical parameters related to this system such as corrosion current density (*i*corr), corrosion potential (*E*corr), and Tafel cathodic coefficient (*β*_c_) were listed in Table 2. The *i*corr were determined from the extrapolation of cathodic Tafel lines, which exhibit a real Tafelian behavior (McCafferty, 2005). The inhibition efficiency (*ɳ*_*Tafel*_ *)* was calculated via using

$$\eta_{Tafel\;}=\left(1-\frac{i_{corr/inh}}{i_{corr}}\right)\times 100,$$ (3)

where *i*_*corr*_ and *i*_*corr/inh*_ represent corrosion current density values without and with Wf-EO, respectively.

**TABLE 2:** Electrochemical parameters generated from PP curves of MS immersed in 1.0 M HCl at different concentrations of Wf-EO at 298 K.

MS/electrolyte interface	*E*_corr_ *mV*_*Ag/AgCl,3M KCl*_	*i*_corr_ *µA* *cm* ^*−2*^	|*β*_c_| *mV dec* ^*−1*^	ɳ _PP_ %
MS/1.0 M HCl	−469.79	611.61	131.1	-
MS/0.1 g L^−1^ of Wf-EO	−414.92	199.54	186.5	67.37
MS/0.3 g L^−1^ of Wf-EO	−418.99	176.2	192.8	71.22
MS/0.5 g L^−1^ of Wf-EO	−405.64	132.4	200.3	78.42
MS/0.8 g L^−1^ of Wf-EO	−395.61	115.40	197.9	81.13
MS/1 g L^−1^ of Wf-EO	−396.12	107.72	191.0	82.39

As can be shown from Figure 4, both cathodic and anodic reactions were inhibited through the addition of Wf-EO in acidic solution. The cathodic parallel Tafel lines (Figure 4) and the slight changes in the cathodic Tafel slopes *β*_c_ (Table 2) indicated that the Tafel’s law was verified in this domain (Rice-Evans et al., 1996). In this sense, the mechanism of H^+^ proton discharge was activation controlled by the addition of Wf-EO in acidic solution without modification of the process mechanism. Nevertheless, in the anodic domain with potentials higher than *−270 mVAg/AgCl, 3M KCl*, Wf-EO did not modify the current *versus* potential characteristics when compared to the uninhibited medium. This phenomenon could be attributed to the desorption of the Wf-EO compounds from the electrode surface at a potential and can therefore be defined as desorption potential *E*_*d*_, commonly reported in the literature (Beniken et al., 2018; Aourabi et al., 2021).

The corrosion potential *E*_*corr*_ is shifted toward more anodic values as shown in Table 2. In this sense, if the displacement in *E*_corr_(inh) is larger than 85 mV from *E*_corr_ in free inhibitor, the inhibitor can be seen as a cathodic or anodic type (Bensouda et al., 2019), and if the displacement in *E*_corr_(inh) is less than 85.00 mV, the inhibitor can be considered as a mixed type (Soltaninejad and Shahidi, 2018; Guruprasad et al., 2019). In this study, the largest value of potential displacement achieved is of the magnitude of *74 mV* indicating that Wf-EO acts as a mixed type inhibitor with the domination of the anodic character.

Table 2 shows that the values of current densities of *i*_*corr*_ decrease considerably with rising Wf-EO concentrations from *611.61 µA cm* ^*−2*^ in 1.0 M HCl alone to *107.72 µA cm* ^*−2*^ at a concentration of 1.00 g L^−1^ of Wf-EO. This diminution in current density provides the inhibition efficiency to reach a maximum value of 82.39%. It can also be noted that no enhancement in the inhibiting efficiency is observed despite increasing the concentration of Wf-EO, that is a plateau of *ɳ*_*Tafe*_ % was registered from 1 g L^−1^ of Wf-EO concentration.

#### EIS Measurements

**FIGURE 5:**
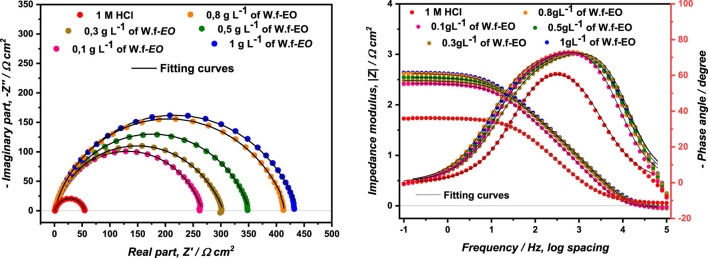
Nyquist and Bode diagrams of the MS in 1.00 M HCl with and without concentrations of Wf-EO at 298 K.

For understanding the mechanism that took place at the electrode/solution interface, EIS constitutes a very powerful technique for investigating corrosion inhibition processes. The Nyquist and Bode diagrams of MS immersed into the corrosive solution 1.0 M HCl, without and with concentrations of Wf-EO are displayed in Figure 5.

The Nyquist diagrams show at first glance only one single depressed capacitive loop, which showed that corrosion of MS can be monitored by the charge transfer process. This phenomenon of capacitance dispersion at the electrode interface strongly depends on the state of the electrode surface, that is, its roughness, degree of polycrystallinity, and also an anion adsorbtion. The deviation from ideal capacitive behavior can be empirically represented by a constant phase element (CPE), a complex impedance having a special property with a phase angle independent of frequency, as reported by Fricke (Fricke, 1932). The impedance of CPE component can be expressed by using

$${\text{Z}}_{\text{CPE}}={\text{Q}}^{-1}{\left(\text{j}\omega \right)}^{-\text{n}},$$ (4)

where *Q* is a related parameter to the electrode capacitance (F s^n−1^ cm^−2^), and *n* is the constant phase exponent often used as a gauge of the roughness or heterogeneity of the surface (0 < *n* < 1) associated with the deviation of the straight capacitive line from 90° by an angle α, whether α can calculated by Eq. 5 and *j* ^*2*^ =−1 is a y number of imaginary whilst *ω* is the angular frequency.

α=90°(1−n). (5)

Therefore, Bode-plots are recommended for the display of EIS data in terms of frequency. Bode-plots permit the detection of regions that are dominated by resistive elements such as resistance of solution *R*_*s*_ and polarization resistance *R*_*p*_ in which a slope of zero was remarked; meanwhile, regions dominated by pure capacitive element for which a slope of (−1) were noticed in the ideal case. However, the width of Gaussian (*φ-log f*) obtained in the presence of different Wf-EO concentrations is much larger than that obtained in 1.0 M HCl alone. This result stipulates most likely the appearance of a second time constant, although not well separated from that of charge transfer in the presence of the different Wf-EO concentrations. This second phenomenon of relaxation or time constant is generally attributed to the presence of an additional obvious process on the metal surface. This simple qualitative analysis confirms the formation of a barrier layer.

**FIGURE 6:**
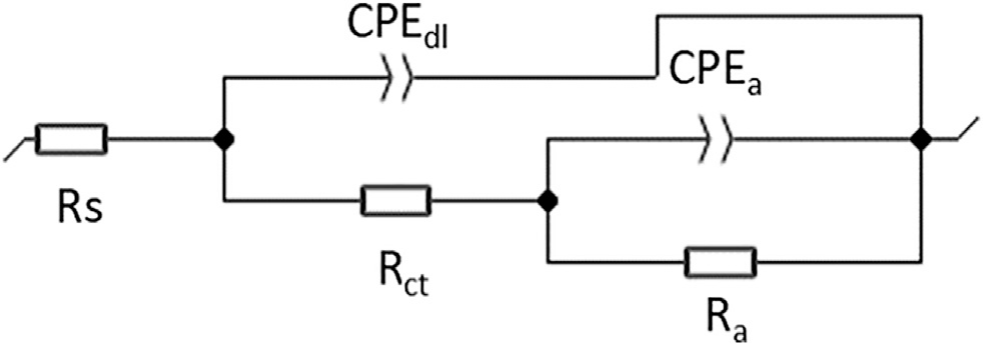
Equivalent electrical circuit model.

Among the numerous equivalent circuits that have been used to describe the present electrochemical interface to extract the necessary parameters for understanding the studied system, only the following circuit with two-time constants *R*_*s*_ *+ CPE*_*dl*_ */[R*_*ct*_ *+ CPE*_*a*_ */R*_*a*_ *]* has been retained as presented in Figure 6. *R*_*s*_ is the solution resistance, *R*_*ct*_ is the charge transfer resistance, and the time constant (*CPE*_*dl*_) at high frequency referring to the charge transfer process. *R*_*a*_ is the resistance of the adsorbed inhibitor whilst *CPE*_*a*_ represents the relaxation time constant of the adsorbed layer to the MS surface. Therefore, an excellent parametric readjustment of the experimental impedance spectra in the Nyquist and Bode planes was obtained. Moreover, the experimental and simulated spectra were well correlated with a χ′^2^/|Z| coefficient, and therefore, they support the validation of this model.

The impedance parameter recorded from the fitting of diagrams and $\eta_{EIS}\%$ are presented in Table 3. The inhibition efficiency issued from the polarization resistance (PR) in the presence of the different Wf-EO concentrations is calculated according to

$$\eta_{EIS}\%=\left(1-\;\frac{R_{p}}{R_{p/inh}}\right)\times 100,$$ (6)

where *R*_*p*_ is the polarization resistance calculated through the following equation (*R*_*P*_ = *R*_*ct*_ + *R*_*a*_).

**TABLE 3:** Inhibition efficiency and EIS parameters for MS in 1.0 M HCl at different concentrations of Wf-EO.

Electrode/electrolyte interface	*R*_*S*_	*Q*_*dl*_	*n*_*dl*_	*R*_*ct*_	*Q*_*a*_	*n*_*a*_	*R*_*a*_	*R*_*p*_	*η*_EIS_%	χ^2^/|Z|
MS/1.0 M HCl	1.16	205.50	0.847	37.23	80.93	0.461	16.53	53.76	--	0.015
MS/0.1 g L^−1^ of Wf-EO	0.94	31.46	0.963	28.35	118.70	0.667	239.60	267.95	79.93	0.037
MS/0.3 g L^−1^ of Wf-EO	0.89	22.50	0.959	39.90	107.50	0.675	267.40	307.30	82.50	0.040
MS/0.5 g L^−1^ of Wf-EO	0.96	27.71	0.943	48.73	100.10	0.678	304.90	353.63	84.79	0.043
MS/0.8 g L^−1^ of Wf-EO	0.93	29.27	0.947	49.81	92.69	0.677	370.90	420.71	87.22	0.049
MS/1 g L^−1^ of W**f-**EO	0.96	27.99	0.936	56.88	83.35	0.674	382.90	439.78	87.77	0.037

R_s_, R_ct_, R_a_, and R_p_ in Ω cm^2^; Q_dl_ and Q_f_ in µF s^n−1^ cm^−2^.

The data displayed in Table 3 clearly shows that in the concentration range, the PR enhances with Wf-EO concentration; hence, the best inhibition efficiency reached was 87.77%. The parameter of *CPE*_*dl*_ component *Q*_*dl*_ decreased abruptly with the addition of Wf-EO when compared to the uninhibited solution through presumably the formation of a protective adsorbed layer. The improvement of this layer was associated with an increase in *n*_*d*_ when compared to the blank solution that was associated with a slight decrease in the surface heterogeneity (Aourabi et al., 2020). It was noted that the charge transfer resistance *R*_*ct*_ increased with increasing concentration of Wf-EO and the total resistance *R*_*p*_ was rather dominated by *R*_*a*_.

According to the model of Bonnel et al. (1983), the resistance associated with the high-frequency portion of the spectrum is hypothesized to contain cathodic and anodic contributions of the charge transfer reaction. This confirms the mixed character obtained from PP measurement. The resistance related to the low-frequency portion is regarded to contain the explicit contribution from mass transport across the porous corrosion products. This latter result was reflected by the lowest value of *n*_*a*_ which can be attributed as a measure of the energy distribution since the adsorbed molecules were electronic charge carriers (Popova and Raicheva, 1996).

#### Adsorption Isotherm

During the process of inhibitor adsorption on the surface of metal - assisted adsorption, the process is controlled by the residual charge on the chemical structure and the inhibitor metal. The increasing addition of a corrosion inhibitor results in a progressive decrease in the rate of corrosion and/or enhancement of corrosion inhibition. The relationship between the concentration of inhibitor and corrosion rate was investigated by several models of isotherms. In this sense, it was reported that the common adsorption isotherms used to adjust the experimental data are Freundlich, Temkin, Langmuir, and the El-Awady kinetic–thermodynamic model. The conventional and linearized forms of isotherms are figured in Table 4.

**TABLE 4:** Linearized and conventional and forms of the most used adsorption isotherm models.

Isotherm	Conventional form	Linearized form		
Langmuir	$\frac{\theta }{1-\theta }=\;K_{ads}C_{inh}$	$\frac{C_{inh\;}}{\theta }=\;\frac{1}{K_{ads}}+\;C_{inh}$		Halambek et al. (2010)
El-Awady	${\left(\frac{\theta }{1-\theta }\right)}^{1/y}=\;K_{ads}C_{inh\;}$	$log\left(\frac{\theta }{1-\theta }\right)=ylogK_{ads}+ylogC_{inh}$		Fu et al. (2011)
Temkin	$exp\left(-2a\theta \right)=\;K_{ads}C_{inh}$	$\theta =\;-\frac{1}{2a}lnK_{ads}-\frac{1}{2a}lnC_{inh}$		Andreani et al. (2016)
Freundlich	$\theta =\;K_{ads}{\left(C_{inh}\right)}^{n}$		$ln\theta =ln\left(K_{ads}\right)+nlnC_{inh}$	Khadom et al. (2010)

**FIGURE 7:**
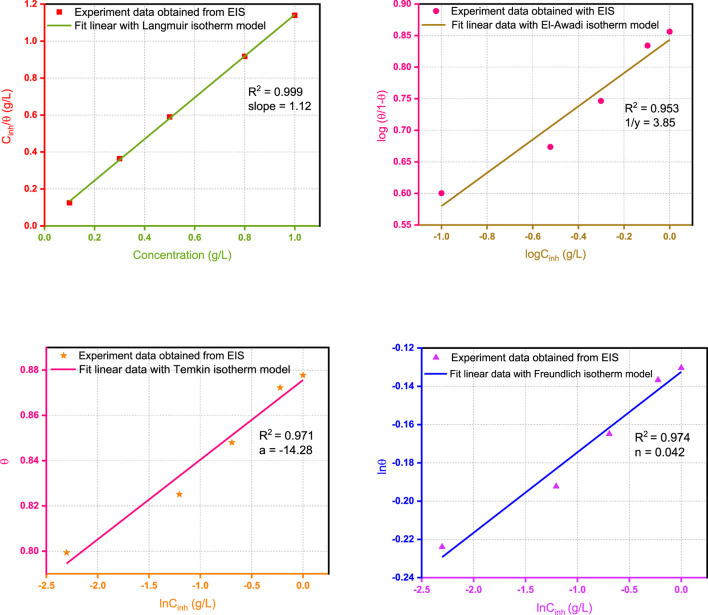
Plots of models of the adsorption isotherm of Wf-EO for MS surface in 1.0 M HCl at 298 K obtained from EIS data.

In the present research work, *θ* values corresponding to different concentrations of Wf-EO are easily determined from EIS (*θ*_*EIS*_) method, by the ratio *η*%/100. Figure 7 displays different models of adsorption isotherm such as Langmuir, Freundlich, Temkin, and El-Awady and also the related parameters of adsorption deduced from these isotherms.

These results show that all the coefficients are close to unity with the better fit provided by the use of the adsorption isotherm Langmuir model with *R* ^*2*^ = 0.9999; meanwhile, the slope of the corresponding formula slightly deviates from unity. Accordingly, the model of Langmuir seems to be more appropriate to describe the adsorption process as reported in earlier works (Durnie et al., 2001; Obot et al., 2009). On the contrary, the reciprocal of ‘*y’* obtained from the El-Awady model is nearly equal to 4 and can therefore suggest that four water molecules were replaced by one from Wf-EO in the inhibition process. Based on this result, it can be concluded that the first is contradictory with the hypothesis on which the Langmuir isotherm is based, which stipulates that one molecule of inhibitor replaces one molecule of water. Moreover, the adsorption process of Wf-EO molecules also follows the Temkin isotherm, which indicated the presence of molecular interaction in the adsorbed layer. However, this hypothesis is practically consistent with the Langmuir isotherm model and it is also confirmed by the negative sign of intermolecular interaction in the adsorption layer ‘*a*’ of the Temkin model (Oguzie, 2007). It is thus fitting that the adsorption process investigated in the present work could not be reasonably modeled by the Freundlich isotherm model even though *R* ^2^ values obtained from the corresponding plots were good.

In summary, the possibility of ideal Langmuir-type adsorption is unthinkable due to fact that the surface heterogeneity is evidenced by EIS measurements where the *CPE* exponents are far from unity. Furthermore, a molecule of water would probably be replaced by three or even four molecules of oil, identical or not, as testified by the El Away isotherm with a possibility of strong interaction between them by reference to the isotherm of Temkin.

## Conclusion

In this work, we shed light on the chemical composition (GC/MS), antioxidant activities, and anticorrosive activity of the essential oil of *W. frutescens* (Wf-EO), and the above analysis and discussion of the experimental results lead to the following main conclusions:

1. Chromatographic analysis (GC/MS) of the essential oils (Wf-EO) revealed that *W. Frutescens* is rich in potentially bioactive compounds with a dominance of β-thujone (26.47%) and comphor (37.86%).
2. A high antioxidant capacity of Wf-EO was confirmed by four tests (TAC, FRAP, DPPH, and beta carotene) even at low concentrations.
3. Wf-EO acts as a good corrosion inhibitor for C steel in 1.0 M HCl solution.
4. PP measurements indicated that the largest potential shift value obtained is in the order of 74 mV indicating that Wf-EO acts as a mixed type inhibitor with the dominance of anodic character.
5. The EIS data showed that the corrosion of C-steel is mainly controlled by charge transfer on a heterogeneous and irregular surface.

## Data Availability

The raw data supporting the conclusions of this article will be made available by the authors, without undue reservation.
